# A Polyphasic Approach to Classification and Identification of Species within the *Trichophyton benhamiae* Complex

**DOI:** 10.3390/jof7080602

**Published:** 2021-07-26

**Authors:** Frederik Baert, Paulien Lefevere, Elizabet D’hooge, Dirk Stubbe, Ann Packeu

**Affiliations:** 1 BCCM/IHEM Fungi Collection, Service of Mycology & Aerobiology, Sciensano, Rue J. Wytsmanstraat 14, B-1050 Brussels, Belgium; Elizabet.Dhooge@sciensano.be (E.D.); Dirk.Stubbe@sciensano.be (D.S.); Ann.Packeu@sciensano.be (A.P.); 2 Service of Mycology and Aerobiology, Sciensano, Rue J. Wytsmanstraat 14, B-1050 Brussels, Belgium; Paulien.Lefevere@sciensano.be

**Keywords:** dermatophytes, Arthrodermataceae, phylogeny, *Trichophyton*, *T. benhamiae*, *T. erinacei*, *T. africanum*, MALDI-ToF, ITS, onchomycosis

## Abstract

In recent years, considerable advances have been made in clearing up the phylogenetic relationships within the Arthrodermataceae family. However, certain closely related taxa still contain poorly resolved species boundaries. Here, we tried to elucidate the species composition of the *Trichophyton benhamiae* species complex using a combined approach consisting of multi-gene phylogenetic analysis based on internal transcribed spacer (ITS) and beta-tubulin (BT) gene regions, morphological analysis, and spectral comparison using MALDI-ToF. We confirmed the existence of 11 different monophyletic clades within the complex representing either species or genetically distinct groups within species. MALDI-ToF spectrometry analysis revealed that most of these clades were readily distinguishable from one another; however, some closely related sister clades, such as *T. europaeum* and *T. japonicum*, were often misidentified as their counterpart. The distinct “yellow” and “white” phenotypes of *T. benhamiae* do not have a clear genetic basis and should thus be considered as different morphotypes of the same species. Strains traditionally considered *T. benhamiae* can be divided into three main clades: (i) *T. benhamiae*, (ii) *T. europaeum*/*T. japonicum*, and (iii) the phylogenetically distant *T. africanum*. While *T. europaeum* and *T. japonicum* are distinguishable based on their genotype, spectral and morphological analysis did not provide clear delimiting characteristics.

## 1. Introduction

Dermatomycoses are one of the most common infections known to humankind; estimates say that 20–25% of individuals will at one point be confronted with a dermatophyte infection [1]. The dermatophytes (Onygenales, Arthrodermataceae) are a group of filamentous fungi that are capable of infecting keratinized tissues of both humans and animals [2]. These infections of the skin, hair, or nail tissues are called dermatophytoses, also commonly known as tinea or ringworm. Ecologically speaking, the dermatophytes can be broadly classified into geophilic, zoophilic, and anthropophilic species [3]. The anthropophilic species naturally occur on human keratinous tissues and have mostly lost the ability to mate sexually. Geophilic species can be found in soil feeding on keratinous debris, while zoophilic species have animals as their natural reservoir.

Due to their clinical relevance, the Arthrodermataceae family has been intensively investigated over the last two centuries. Taxonomy based on phenotypic criteria, such as in vitro morphology, clinical symptoms, and physiology, gave rise to the description of many species; however, the true phylogeny of the dermatophytes only truly came into focus with the advent of molecular techniques. Classification based on genetic markers such as internal transcribed spacer (ITS), beta-tubulin (BT), and translation elongation factor-1-alfa (TEF1a) has clarified much of the dermatophyte taxonomy [4,5,6,7]. Multilocus phylogenetic analysis by de Hoog et al. eventually solidified the existence of seven genera, namely: *Trichophyton*, *Epidermophyton*, *Nannizzia*, *Microsporum*, *Paraphyton*, *Lophophyton*, and *Arthroderma* [5]. While these genetic markers have been remarkably successful at delineating most species, complexes of closely related species still exist around some *Trichophyton* species, such as *T. mentagrophytes* and *T. benhamiae*.

The *Trichophyton benhamiae* complex traditionally contained six closely related species: the zoophilic species *T. benhamiae*, *T. erinacei*, *T. eriotrephon*, *T. verrucosum*, and *T. bullosum* and the antropophilic species *T. concentricum*. Earlier phylogenetic studies have shown that most of these species are reasonably well-defined; *Trichophyton benhamiae* itself, however, seems to be paraphyletic [5,8,9]. *Trichophyton benhamiae* is a zoophilic species that can cause highly inflammatory tinea corporis and tinea capitis in human hosts [10,11,12]. The most common host is the guinea pig (*Cavia porcellus*) and human infection is often linked to contact with these animals [10,11,12]. The species was first described as *Arthroderma benhamiae* and was considered the perfect state of *T. mentagrophytes* [13]. Takashio (1974) later observed the existence of two separate races within *T. benhamiae*, an ‘Americo-European’ race and an ‘African’ race, through the use of mating experiments and phenotypic analysis [14]. Molecular data has shown that these races are quite genetically distant from each other and that they are not monophyletic. Čmoková et al. recently proposed the name *T. africanum* to cement the African race of *T. benhamiae* as a separate species, together with two other new species within the *T. benhamiae* species complex: *T. europaeum* and *T. japonicum* [15]. Within the ‘Americo-European’ race, two phenotypic variants can be distinguished: the white and the yellow phenotype. While the white phenotype has been around since the species was first described, the yellow variant was only first discovered in 2008 [16]. Genetically, these phenotypic variants seem to be indistinguishable [5,8,15]. In the last few decades, *T. benhamiae* has also become a more prevalent agent of dermatophyte infection in several European regions [16,17,18,19]. It is striking to see that human infection by *T. benhamiae* was barely on the radar a decade ago, while it is becoming more and more commonplace nowadays [2,18,19]. Recently, terbinafine-resistant strains of *T. mentagrophytes* have been isolated, showing that antifungal resistance is an emerging threat among dermatophytes [20]. This underlines the importance of correct taxonomic identification, even though at present no difference in antifungal susceptibility has been observed among species from the *T. benhamiae* complex [21].

Using a polyphasic approach consisting of multilocus phylogenetic analysis, MALDI-ToF mass spectroscopy, and morphological analysis, this study aims to clarify the species boundaries in the *T. benhamiae* complex and to provide clear distinguishing characteristics for the identification of these species in the lab.

## 2. Materials and Methods

### 2.1. Isolates and Morphological Analysis

In this study, a total of 182 strains of the BCCM/IHEM fungi collection belonging to the *T. benhamiae* species complex were analyzed. An overview of the origin and history of these strains can be found in supplementary Table S1. Macro- and microscopic features of the isolates were analyzed after incubation for 7–21 days at 25 °C on diluted (1/10) Sabouraud dextrose agar (S10) and on Harold’s agar (M40Y).

### 2.2. DNA Extraction, PCR and Sequencing

Strains were cultured on Sabouraud dextrose broth for five days. Some slow-growing isolates were incubated until enough biological material for analysis could be obtained. Genomic DNA of the strains was extracted using the Invisorb Spin Plant Mini Kit (Invitek, Berlin, Germany). The extraction kit was used according to the manufacturer’s instructions, with some adaptations: (1) before lysis, a lyophilization step and subsequent bead beating was added to facilitate the disruption of the fungal cell wall, and (2) the lysis time was raised to more than 2 h.

Two gene regions of the genomic DNA were amplified and sequenced: (1) the primers Bt2b and Bt2a described by Glass and Donaldson [22] were used for amplification and sequencing of the partial β–tubulin (BT) gene, and (2) the ITS regions were amplified using primers ITS5 and ITS4 [23]. BT was chosen as a secondary region since it has been shown to provide the highest resolution when determining clades in the dermatophyte family when choosing among the most commonly used markers, with the exception of the barcoding regions ITS [5,24].

PCR amplicons were purified using ExoSAP-IT PCR Product Cleanup (Affymetrix, Santa Clara, CA, USA). Sanger sequencing was performed with an ABI 3130xl Genetic Analyzer (Applied Biosystems, Waltham, MA, USA). Sequences generated in this study were deposited to the European Nucleotide Archive (ENA) under the accession numbers OU230982-OU231104 and OU231117-OU231239.

Consensus sequences were assembled and edited with DNASTAR lasergene 10 (DNASTAR, Madison, WI, USA). Using BT and ITS sequences, a multi-locus phylogenetic analysis was performed involving all 182 strains. The multiple sequence alignment was constructed with MAFFT version 7.394 using the FFT-NS-i iterative refinement method. The scoring matrix for nucleotide sequences was set to 1PAM/κ = 2. Afterwards, the alignment was manually assessed and checked for inconsistencies. On the basis of this dataset, a maximum likelihood (ML) phylogeny was constructed using IQTree version 1.6.12 [25]. Branch support was calculated using 1000 bootstrap replicates. The dataset was subdivided into 4 gene partitions: ITS1+ITS2, 5.8S+28S, BT introns, and BT exons. Modelfinder was used to determine the best-fit model for each partition [26].

### 2.3. MALDI-ToF Spectroscopy

Strains were cultured on Sabouraud Chloramphenicol (SC) agar plates at 25 °C for 3 days. For some slow-growing isolates, the incubation time was extended until enough biological material for analysis could be obtained. Strains used for the creation of the reference database were cultured on five SC plates, four for the creation of main spectrum profiles (MSPs), and one for quality control. For species-level identification, one SC plate was used per isolate. The strains used for species-level identification of *Trichophyton verrucosum* were incubated on one plate of Casein agar enriched with thiamine, inositol, and chloramphenicol at 25 °C.

The protein extractions were prepared according to the method outlined by Normand et al. with some modifications. The samples were first suspended in 300 µL R.O. (reverse osmosis, Satorius, Goettingen, Lower Saxony, Germany) water and vortexed, before adding 900 µL ethanol absolute (dehydrated) [27]. After removal of the hydro-alcoholic solution, the pellet was air-dried, and finally 50 µL 70% formic acid and 50 µL acetonitrile were subsequently added following a final vortex step. 

A Microflex LT MALDI-ToF mass spectrometer (Bruker Daltonics, Bremen, Germany) with default settings was used to acquire mass spectra. Data were then exported into MALDI Biotyper v2.1 (Bruker Daltonics) software. A database of MSPs was constructed using strains representative of each species/clade that was distinguishable on the basis of the phylogenetic analysis; strains used for the construction of this database are indicated with a “*” in Supplementary Table S1. For each reference strain, four MSPs were created, merging ten mass spectra from one subculture using the MSP creation function included in the MALDI Biotyper v2.1 software.

For species-level identification of the isolates, one extraction was used to acquire four mass spectra replicates. The MS data of each of these replicates were then matched to the reference database, resulting in the designation of a “best match” based on the log score value calculated by the Biotyper software v2.1. An identification was deemed valid when log scores were ≥1.7 and at least three out of the four replicates were identified as the same species.

For the creation of three-dimensional principal component analysis (PCA) plots, spectra were processed using the R package MALDIquant for R v3.6.3 using the workflow outlined by Gibb and Strimmer (2017) [28,29,30]. Peak lists of the mass spectra were subsequently loaded into the open access software Mass-Up v1.0.14 for peak matching and the creation of PCA plots [31]. Peaks were matched using both intra- and intersample matching (MALDIquant, tolerance = 0.002), and the PCA was subsequently generated using the parameters (i) max components = −1, (ii) variance covered = 0.95, (iii) normalize, and (iv) discretize.

## 3. Results

### 3.1. Phylogenetic Analysis

**Figure 1:**
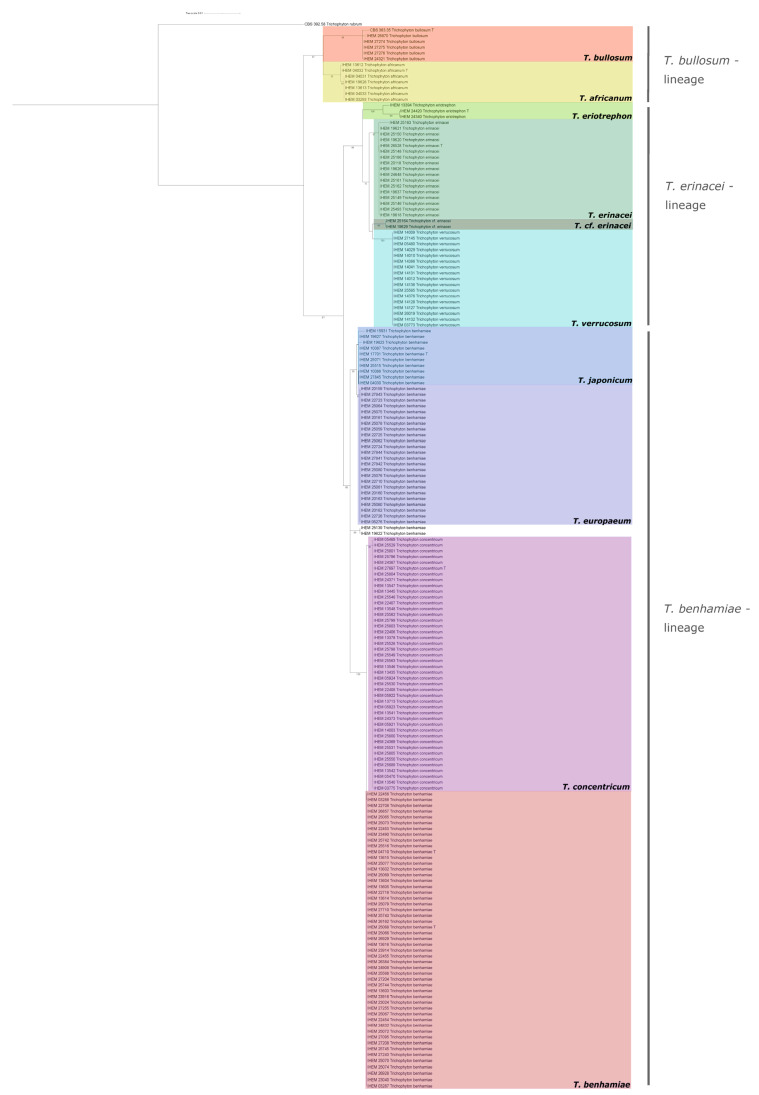
Maximum likelihood phylogenetic tree of the *Trichophyton benhamiae* complex based on internal transcribed spacer (ITS) and beta-tubulin (BT), with the type strain of *Trichophyton* rubrum (CBS 392.58) as the outgroup. Tree inferred via maximum likelihood method using IQ-TREE software. Bootstrap values are provided at the nodes based on 1000 bootstrap replicates, only values higher than 70% are displayed. *Trichophyton rubrum* CBS 392.58 was used as outgroup, type strains are indicated with “T”.

The phylogenetic tree constructed based on the concatenated ITS+BT multiple sequence alignment divides the complex into three major lineages within the *T. benhamiae*-complex, namely *T. benhamiae*-, *T. erinacei*-, and *T. bullosum*-lineages (Figure 1). The monophyly of the species *T. erinacei*, *T. eriotrephon*, *T. verrucosum*, *T. bullosum*, *T. africanum*, and *T. concentricum* is strongly supported. 

#### 3.1.1. *T. benhamiae* Lineage

The strains traditionally considered to belong to the ‘Americo-European race’ of the species *T. benhamiae* clustered into two separate groups, here indicated as the *T. benhamiae* clade containing strains of both *T. benhamiae* var. *benhamiae* and *T. benhamiae* var. *luteum*, and the *T. japonicum*/*europaeum* clade (Figure 1). 

The first group contained a paraphyletic cluster of *T. benhamiae* strains positioned next to a highly supported clade of *T. concentricum*. We consider this paraphyletic clade *T. benhamiae* sensu strictu as it contains the original type strain for *T. benhamiae*. Between *T. benhamiae* s.s. and *T. concentricum*, two distinctive SNPs can be defined in the ITS alignment at positions 73 of ITS1 and position 94 of ITS2. The *T. benhamiae* s.s. clade contained all the yellow phenotype (*T. benhamiae* var. *luteum*) strains, but also regular white phenotype (*T. benhamiae* var. *benhamiae*) strains. No clear genetic difference can be defined to distinguish between the two phenotypes. 

The second clade was a sister clade to the *T. benhamiae* clade and was phylogenetically quite close, but clearly distinct from it. This clade contained both *T. europaeum* and *T. japonicum*, which are very closely related. The *T. europaeum* clade receives high support; the *T. japonicum* clade, however, does not. Only one distinctive SNP exists between *T. europaeum* and *T. japonicum* at position 155 of ITS1; BT does not contain any SNPs with predictive power for these two species. 

Strains IHEM 25139 and IHEM19622 belonged to a separate well-supported clade and contain 5 SNPs in the ITS regions when compared to *T. europaeum* and *T. japonicum*.

#### 3.1.2. *T. erinacei* Lineage

The *T. erinacei* clade contained three well defined species with high support: *T. erinacei*, *T. verrucosum*, and *T. eriotrephon*. Characteristic SNPs for *T. erinacei* can be found at positions 217 of ITS1 and positions 59 and 95 of ITS2. *Trichophyton verrucosum* can be recognized on the basis of SNPs at the positions 40, 129, 130, and 210 of ITS, 14 and 188 of ITS2, and position 12 of BT. Two strains, IHEM 19629 and IHEM 25164, form a well-supported sister clade to *T. verrucosum*. These strains display some, but not all, of the SNPs that are typical for *T. verrucosum*, but also contain some unique SNPs at positions 236 of the ITS1 region and 141 of the 5.8S ribosomal RNA. For the remainder of this paper, these strains will be referred to as *T.* cf. *erinacei*, named according to their high morphological resemblance to *T. erinacei*.

#### 3.1.3. *T. africanum* Lineage

At the base of the phylogenetic tree, the strains known to be of the ‘African race’ are clustered together in a well-supported monophyletic sister clade of *T. bullosum*. This result supports the notion that the ‘African race’ of *T. benhamiae* should be considered a separate species, the recently described *T. africanum* [15].

### 3.2. Morphology and Ecology

**Figure 2:**
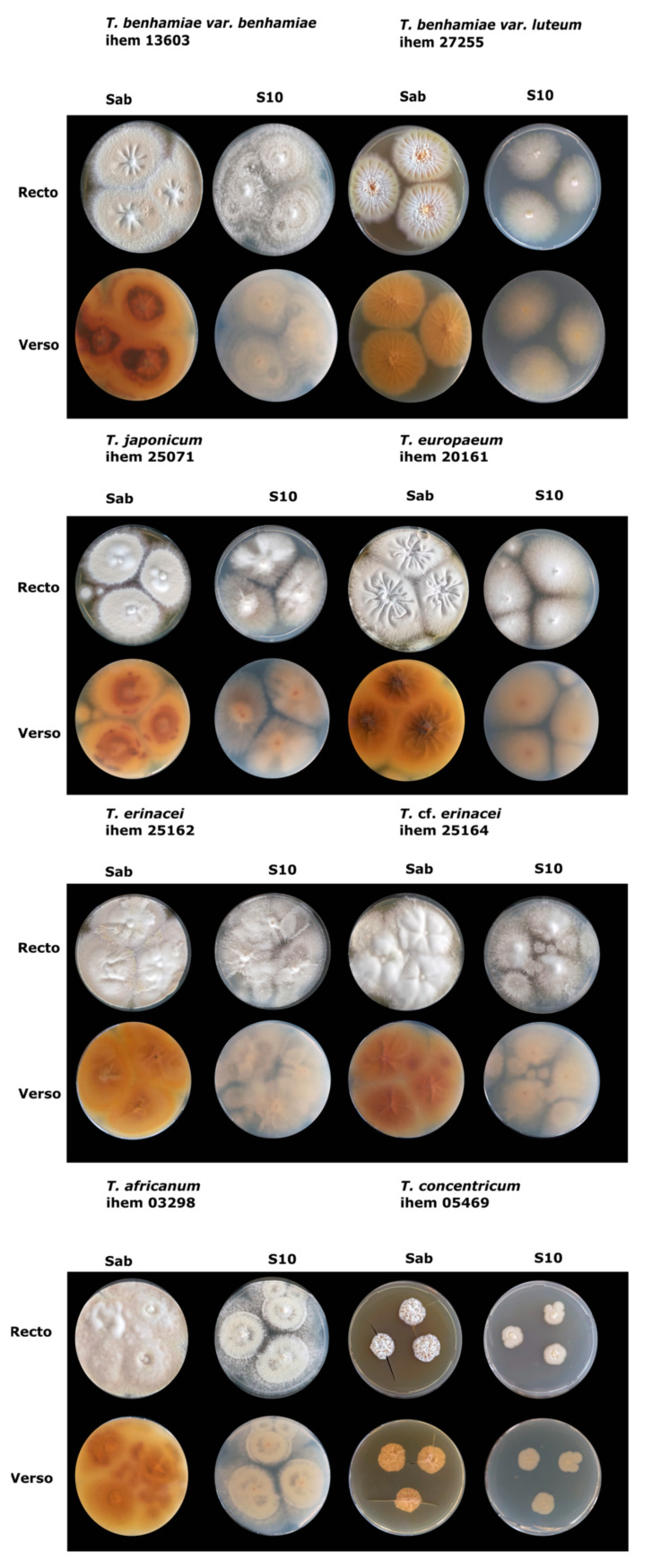
Pictures illustrating the macromophology of strains representative of *T. benhamiae* var. *benhamiae* (IHEM 13603), *T. benhamiae* var. *luteum* (IHEM 27255), *T. japonicum* (IHEM 25071), *T. europaeum* (IHEM 20161), *T. erinacei* (IHEM 25162), *T.* cf. *erinacei* (IHEM 25164), *T. africanum* (IHEM 03298), and *T. concentricum* (IHEM 05469). Isolates were incubated for 14 days on Sabouraud (Sab) and diluted Sabouraud agar (S10).

*Trichophyton benhamiae* s.s. consists of two phenotypic variants mainly characterized by their white (var. *benhamiae*) or yellow (var. *luteum*) color on Sabouraud agar plates (Figure 2). Furthermore, *T. benhamiae* var. *benhamiae* formed colonies with a brown to beige, and sometimes red, reverse colony color, cottony to powdery, sometimes fluffy mycelium, and often a tendency to pleomorphise on Sabouraud agar. *Trichophyton* var. *benhamiae* also showed a high production of pyriform and round microconidia. *Trichophyton benhamiae* var. *luteum,* on the other hand, showed a bright yellow to orange colony color and a much lower growth rate then var. *benhamiae*. No microconidia were observed on Sabouraud agar medium, but on MY40 many round microcondia sporulated, often arranged in clusters. Strains from both variants were mostly sampled from guinea pigs or humans who had been in contact with guinea pigs. 

*Trichophyton concentricum* is the only antropophilic species in the *T. benhamiae* complex and was thus only sampled from humans (Table 1); the species is endemic to southeast Asia. This species is mainly characterized by its slow growth and smooth to fluffy colony texture often with a cerebriform or crater-like aspect. Colony color was mostly white to beige, rarely pink or orange. Sporulation was rare on all media, and chains of chlamydospores were observed.

Strains of the *T. europaeum*-*japonicum* clade showed a white to cream colony color at the surface, while the reverse was orange or brown, sometimes red. Colony texture was cottony to powdery, and sporulation was abundant with round to pyriform microconidia. No clear morphological characteristics were observed that are able to distinguish between these two species. What is more, no clear morphological differences could be seen between these two species and the white variant of *T. benhamiae*. The strains examined here were all of European origin and mostly isolated from guinea pigs and human patients, except for IHEM 4030 which was sampled from a dog (Table S1).

*Trichophyton erinacei* is a common zoophilic species found in European hedgehogs (*Erinaceus europaeus*) and the African four-toed hedgehog (*Atelerix albiventris*). Examined strains of *T. erinacei* had a white to yellow surface color and a yellow or orange to brown reverse. Colony texture was powdery, sometimes cottony. High amounts of round and pyriform microconidia were produced on Sabouraud agar and MY40. The strains designated *T.* cf. *erinacei* in this study showed the same phenotype as *T. erinacei*. 

*Trichophyton verrucosum* is a zoophilic species associated with cattle that often causes ringworm infections in humans. On agar plates, it is a slow growing species forming smooth colonies. Surface colony color was white or, rarely, yellow, while the reverse was yellow to orange. Most strains showed no sporulation, but sometimes pyriform microconidia were observed, and intercalated and terminal chlamydospores were often present. 

*Trichophyton africanum*, previously known as ‘African-race’ *T. benhamiae*, is a rare, or at least under-sampled, species originating from Africa. Two of the examined strains were discovered in Belgium on human skin and nails. The most recent strain in collection was isolated in 1978, so little is known about the current status of this species in the wild. Both the obverse and reverse colony color were a shiny yellow, sometimes with orange or brown. Round and pyriform microconidia were abundant on both Sabouraud agar and MY40. The genetically close *T. bullosum* is another rare species and is associated with horses and donkeys. The species is characterized by a very slow growth and a transparent, pale yellow colony color. Polymorphic filaments with intercalated and terminal chlamydospores were observed in all isolates.

### 3.3. MALDI-ToF Spectrometry

Table 1 shows the results of database comparison analysis of the MALDI-ToF spectra. Misidentifications were mostly limited to closely related species, evidenced by the combined scores per clade in Table 1. *Trichophyton europeaum* and *T. japonicum* were not distinguishable with high confidence; correct identification was achieved in 50 to 70% of cases, respectively. When considering these two species as one clade, however, accuracy rose to 82%. A similar result was observed when examining the *T. benhamiae* clade, containing *T. benhamiae* var. *benhamiae*, and *T. benhamiae* var. *luteum*, and between the strains designated *T.* cf. *erinacei* and the species *T. erinacei*. The species *T. africanum*, *T. bullosum*, and *T. concentricum*, however, were well defined and all received correct identifications in more than 86% of cases. For three strains, log scores were too low for identification using MALDI-ToF MS, namely IHEM 14011 (*T. verrucosum*), IHEM 25530 (*T. concentricum*), and IHEM 25563 (*T. concentricum*).

**Figure 3:**
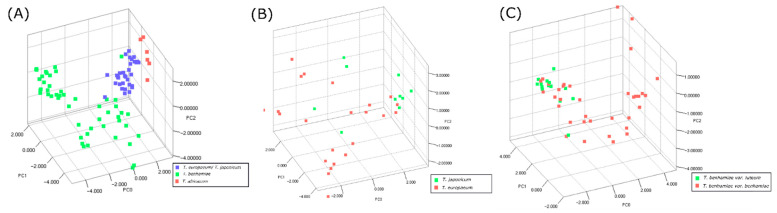
Principal component analysis (PCA) 3D visualization of the three first components made in MassUp. (**A**) Using the strains traditionally designated *T. benhamiae* (*T. africanum*, *T. europaeum*, *T. japonicum*, *T. benhamiae*); (**B**) using strains designated *T. europaeum* and *T. japonicum* based on ITS genotype; (**C**) using strains designated *T. benhamiae* var. *benhamiae* and *T. benhamiae* var. *luteum* based on morphology.

PCA analysis of the spectra shows quite well-defined clusters when considering the clades *T. benhamiae*, *T. europaeum*/*japonicum*, and *T. africanum* (Figure 3). Further subdivision of the *T. benhamiae* and *T. europaeum*/*japonicum* clades, however, does not result in clearly divided clusters, consistent with the results of the database identifications (Figure 3).

## 4. Discussion

Phylogenetic analysis of the ITS and BT gene regions provides strong support for the three major lineages within the *T. benhamiae* complex, namely *T. benhamiae*, *T. erinacei* and *T. bullosum* lineages. Within these lineages, most species were well defined and supported with high bootstrap values, although delineation of the recently introduced species *T. europaeum* and *T. japonicum* proved difficult using these markers (Figure 1). *T. europaeum* and *T. japonicum* have been shown to be more easily separated phylogenetically when using microsatellite markers and the Glyceraldehyde-3-Phosphate Dehydrogenase (*GAPDH*) gene [15]. While BT did not differ between these two species, ITS showed one informative SNP and can thus be used as a barcoding sequence. In practice, however, due the limited number of isolates currently analyzed for these species, and the high possibility of incomplete lineage sorting in recently diverged species such as *T. europaeum* and *T. japonicum*, caution is advised when using ITS as the only marker. This was recently revealed to be the case for *T. tonsurans* and *T. equinum* [32]. The usage of mating-type (*MAT*) loci of these closely related species seems to be the most unambiguous way to delineate them; however, a polyphasic approach is advised [15,32]. 

The ITS genotype of strains IHEM 25139 and IHEM 19622 was close to that of *T. europaeum* and *T. japonicum* but contained 5 SNPs. On the basis of two of these substitutions being critical for the differentiation of *T. benhamiae* and the *T. europaeum*/*T. japonicum* clade, Cmokova et al. suggested that IHEM 25139 could be a hybrid between these species; however, the other three SNPs are not present in either *T. benhamiae* or *T. europaeum*/*T. japonicum* [15]. The strains representing this genotype are quite old (both were isolated in 1963) and, to our knowledge, similar strains have not been isolated since then. Further population studies would be useful to determine the relative prevalence of this genotype, if it still exists at all in the wild, and if its ecology is any different to other clades within the *T. benhamiae* species complex. 

Although morphologically and ecologically closest to *T. erinacei*, phylogenetic analysis placed the *T.* cf. *erinacei* strains closest to *T. verrucosum*. These two strains were sampled from four-toed hedgehogs (*Atelerix albiventris*) in the Ivory Coast between 1974 and 1975 and were described by Gregory et al. as having a faster growth rate and paler yellow reverse then *T. erinacei* from European hedgehogs [33]. A Blastn search (Genbank) revealed that several strains of diverse origins shared this ITS genotype (Table 2). However, one SNP at position 141 of the 5.8S rRNA region was unique to the *T.* cf. *erinacei* strains described in this paper. The most recent strain from the Blastn search was sampled from a European hedgehog in 2017. Kargl et al. saw a link between host species and ITS genotype. They isolated several strains from European hedgehogs of the divergent *T.* cf. *erinacei* genotype [34]. Kargl et al. concluded that the divergent ITS genotype of the *T.* cf. *erinacei* clade was linked to the host species being European hedgehogs as opposed to African four-toed hedgehogs. However, *T.* cf. *erinacei* strains IHEM 19629 and IHEM 25164 cast doubt on that hypothesis since they were isolated from four-toed hedgehogs. Moreover the type strain (IHEM 26528) of *T. erinacei* was isolated from a European hedgehog. Strain 0912m230081 was isolated from a beard sycosis, but no details are known about animal contact; the strain was originally identified as *T. eriotrephon* [19]. The oldest strain known is ATCC 24552 which was isolated in Canada from a mouse. *T.* cf. *erinacei* strains seem to be quite rare but have been isolated in Africa, Europe, and North America during the past few decades [19,34]. Screening of 412 European hedgehogs at a wildlife center in Paris did not yield any strains of this genotype [35]. 

Identification to species level using MALDI-ToF MS was highly accurate for the species *T. benhamiae* (94% not considering variants), *T. concentricum* (98%), *T. africanum* (86%), *T. bullosum* (100%), and *T. verrucosum*.

Differentiating between *T. japonicum* and *T. europaeum* proved more difficult. The identification accuracy of MALDI-ToF MS for these species was less than ideal; only 50% of *T. europaeum* and 70% of *T. japonicum* strains were correctly identified. The species have a very similar protein profile, since most misidentifications were of each other. This is reflected in a much higher accuracy of identification when considering them a single taxon, at 82%.

While differing significantly in their phenotypic appearance, *T. benhamiae* var. *benhamiae* and *T. benhamiae* var. *luteum* did not have any distinguishing differences in their ITS and BT gene sequences. Identification accuracy of the variants of *T. benhamiae* was decent at 74% for *T. benhamiae* var. *benhamiae* and 84% for -var. *luteum*. In order to differentiate between these variants, a simple morphology check remains advisable. When combined, *T. benhamiae* was quite distinct from other species, resulting in a 94% correct identification rate. The closely related *T. concentricum* is quite easily distinguishable from *T. benhamiae* using MALDI-ToF, as only one misidentification was observed. *Trichophyton erinacei* isolates were usually well identified; wrong identifications for this species arose because of confusion with *T.* cf. *erinacei*. *Trichophyton* cf. *erinacei* isolates were identified correctly; however, due to the low sample size, this result is still tentative. Due to the higher degree of taxonomic complexity introduced by recently described species within the complex, the accuracy of identification was somewhat lower in this study compared to previous studies [9,36,37]. This is illustrated by the fact well-established species like *T. concentricum*, *T. benhamiae*, and *T. bullosum* all received identification scores higher than 86%. To our knowledge, this is the first study in which the species *T. africanum*, *T. japonicum*, *T. europaeum*, and both variants of *T. benhamiae* have been considered separate entities for MALDI-ToF-based identification. MALDI-ToF MS remains a fast and reliable technique for the identification of dermatophytes of the *T. benhamiae* complex to the species level, although caution is advised when dealing with very closely related species such as *T. japonicum* and *T. europaeum* as the chance of cross-identification is high. 

In conclusion, the combined use of ITS and BT sequencing, MALDI-ToF spectrometry, and morphological analysis provides strong defining characteristics for the species *T. benhamiae*, *T. concentricum*, *T. erinacei*, *T. verrucosum*, *T. bullosum*, *T. africanum*, and the *T. japonicum*/*T. europaeum* clade. Differentiation between *T. japonicum* and *T. europaeum* proved to be difficult in this study, the main defining characteristic between these species being a single SNP in the ITS gene. Other molecular techniques such as *GAPDH/MAT* gene sequencing and the use of micro-satellite markers have been shown to provide more definition [15].

## Figures and Tables

**Table 1:** Identification results of *T. benhamiae* complex species using MALDI-ToF mass spectroscopy. An identification was deemed valid when log scores were ≥1.7 and at least three out of the four replicates were identified as the same species, strains for which any of these criteria were not met were counted in the column ‘no identification’. When an identification did not match the identity based on ITS+BT genetical analysis, the identification was deemed an ‘incorrect identification.’ For certain clades (in bold) of closely related species/variants combined scores were calculated where identification was considered correct if the strain was identified as either one of the species/variants comprised in the clade.

Clade/Species	Number of Samples			
	Total	Correct Identification	Incorrect Identification	No Identification
***T. europaeum*** **-*japonicum* clade**				
*T. europaeum*	24	12 (50%)	6 (1 *T. benhamiae*, 1 *T. erinacei*, 4 *T. japonicum*)	6
*T. japonicum*	10	7 (70%)	2 (1. *T. benhamiae*, 1 *T. erinacei*)	1
**Combined**	34	28 (82%)	4 (2 *T. benhamiae*, 2 *T. erinacei*)	2
***T. benhamiae*** **clade**				
*T. benhamiae* var. *benhamiae* (white phenotype)	35	26 (74%)	7 (5 *T. benhamiae* var. *luteum*, 1 *T. africanum*, 1 *T. concentricum*)	2
*T. benhamiae* var. *luteum* (yellow phenotype)	16	13 (81%)	2 (2 *T. benhamiae*)	1
**Combined**	51	48 (94%)	2 (1 *T. africanum*, 1 *T. concentricum*)	1
*T. africanum*	7	6 (86%)	1 (1 *T. benhamiae*)	0
*T. bullosum*	2	2 (100%)	0	0
*T. concentricum*	43	42 (98%)	0	1
*T. verrucosum*	7	6 (86%)	0	1
***T. erinacei*** **clade**				
*T. erinacei*	17	11 (64%)	4 (4 *T.* cf. *erinacei*)	2
*T.* cf. *erinacei*	2	2 (100%)	0	0
**Combined**	19	18 (95%)	0	1

**Table 2:** Overview of sequences on genbank of similar ITS genotype as *T.* cf. *erinacei* strains IHEM 19629 and IHEM 25164.

Genbank Accession nr.	Name	Place of Origin	Source	Isolation Date
MF153407.1	DSM 104923	Germany	European hedgehog (*Erinaceus europaeus*)	2017
KY885208.1	0912m230081	France	Human beard	2009
EU181452.1	NCPF 431	Lyon, France	Hedgehog (presumed European)	1974
KJ606083.1	ATCC 24552	Canada	Mouse (*Mus musculus*)	1972

## Data Availability

The data presented in this study are openly available in the European Nucleotide Archive (ENA) at http://www.ebi.ac.uk/ena/data/view/OU230982-OU231104 (accessed on 22 June 2021) and http://www.ebi.ac.uk/ena/data/view/OU231117-OU231239 (accessed on 22 June 2021).

## Supplementary Materials

The following are available online at https://www.mdpi.com/article/10.3390/jof7080602/s1, Table S1: overview of background information of strains used in this study.
